# Metagenomic profiling reveals shared resistome signatures between humans and pigs in Vietnamese smallholder farms

**DOI:** 10.1038/s44259-026-00223-6

**Published:** 2026-06-02

**Authors:** Le Thi Kieu Linh, Truong Nhat My, Nhat Thi Tran, Le Huu Song, Dennis Nurjadi, Sébastien Boutin, Thirumalaisamy P. Velavan

**Affiliations:** 1 Institute of Tropical Medicine, Universitätsklinikum Tübingen and German Centre for Infection Research (DZIF), Tübingen, Germany; 2 Vietnamese - German Centre for Medical Research (VG-CARE), Hanoi, Vietnam; 3 108 Military Central Hospital, Hanoi, Vietnam; 4 National Institute of Veterinary Research, Hanoi, Vietnam; 5 University of Lübeck, Institute of Medical Microbiology, Lübeck, Germany; 6 University Hospital Schleswig-Holstein, Campus Lübeck, Institute of Medical Microbiology, Lübeck, Germany; 7 German Center for Infection Research (DZIF), Partner Site Hamburg-Lübeck-Borstel-Riems, Hamburg-Lübeck-Borstel-Riems, Lübeck, Germany; 8 University Hospital Schleswig-Holstein, Campus Kiel, Institute of Medical Microbiology, Kiel, Germany; 9 Airway Research Center North (ARCN), Member of the German Center for Lung Research (DZL), Lübeck, Germany; 10 Faculty of Medicine, Duy Tan University, Da Nang, Vietnam

**Keywords:** Genetics, Microbiology

## Abstract

Antimicrobial resistance (AMR) is a global health concern, yet the extent of resistant genes and microbial exchange between humans and livestock in low- and middle-income countries remains underexplored. Vietnam, an AMR hotspot, was studied using shotgun metagenomic sequencing of paired faecal samples from pigs and caretakers across 50 small-scale farms. Results revealed 10,270 antimicrobial resistance genes (ARGs) representing 550 unique types, including clinically relevant *mcr*, *bla*_OXA-58,_ and *optrA* genes. Pigs showed higher total AMR abundance, while workers harboured richer resistomes. Approximately 52% (288/550) of ARGs were shared between hosts, dominated by aminoglycoside, β-lactam, and tetracycline resistance genes, often co-located with mobile genetic elements, indicating horizontal transfer potential. Closely related *Escherichia coli* strains were identified in both hosts, consistent with strain sharing or exposure to common sources beyond individual farms. These findings highlight the human–pig interface as an important setting for shared AMR signatures and support the need for integrated One Health surveillance and antimicrobial stewardship.

## Introduction

Antimicrobial resistance (AMR) has emerged as one of the greatest global health crises, threatening human health, food security, and sustainable development. In 2019, an estimated 4.95 million deaths were associated with bacterial AMR, including 1.27 million directly attributable to resistant infections and by 2050, this toll is projected to rise to 8.22 million^1^. Vietnam is recognised as a hotspot for AMR, with high prevalence in both human and animal sectors, underscoring the need for country-level studies. AMR in bacteria arises mainly by two processes: genetic mutations affecting antibiotic targets and by horizontal transfer of antimicrobial resistance genes (ARGs) carried on mobile genetic elements (MGEs)^2^. Occupational exposure provides a distinct setting for the transmission of both commensal and pathogenic bacteria between animals and livestock workers, occurring either through direct^3^ or indirect contact^4^. As a result, overlapping resistance patterns have been documented between livestock and their caretakers on farms^5^. Beyond clinical settings, agriculture, the environment, and communities serve as reservoirs that facilitate the selection of resistant bacteria and genes, thereby increasing the risk of transmission to the humans^6^.

The use of antibiotics, even at subtherapeutic levels, can reshape and expand the gut resistome. Globally, animals accounted for approximately 73% of total antibiotics consumption in 2017, and with usage projected to raise further by 8% by 2030^7,8^. In Vietnam, antimicrobial consumption was approximately 3838 tonnes in 2015, with livestock accounting for 71.1% of total use; pigs alone represented 41.7%^9^. Practices such as mislabelled premixed feeds^10^, unrestricted over-the-counter antibiotic sales^11^, and the use of clinically important antimicrobials in livestock^12^ exacerbate the problem, while hindering efforts to accurate quantification of antibiotic use and its impact on AMR.

Despite this heavy antibiotic reliance, integrative studies examining the microbiome and resistome at the human–livestock interface in Vietnamese smallholder farming systems remain limited. Previous work in Vietnam and other LMIC settings has provided important insights into gut and environmental resistomes, including the high background prevalence of clinically relevant resistance genes in communities and the broader interconnectedness of human, animal, and environmental AMR reservoirs^13,14^. However, fewer studies have combined paired sampling of pigs and their caretakers from the same farms with deep shotgun metagenomics and strain-level analysis to assess resistome overlap, shared genetic contexts, and host-associated microbial relatedness in this specific setting. Metagenomic approaches provide a powerful and unbiased framework for such analyses, enabling comprehensive characterisation of AMR abundance, diversity, and genomic context beyond culture-based or targeted methods^15^. To address this gap, we performed deep metagenomic sequencing of paired faecal samples from pigs and livestock caretakers to provide an integrated overview of resistance profiles across hosts, offering new insights into resistome overlap and strain relatedness at the human–animal interface.

## Results

### Validation of Controls

Sequencing of the negative control yielded 4270 reads, none of which were taxonomically classified. The mock microbial community was profiled as expected. No unexpected taxa were detected in the positive control, except for trace amounts of *Bacillus* species (<0.1%), likely representing bioinformatic artefacts. A comparison between the observed and theoretical relative abundances is shown in Supplementary Fig. 1.

### Resistome characterization in pigs and livestock workers

**Fig. 1:**
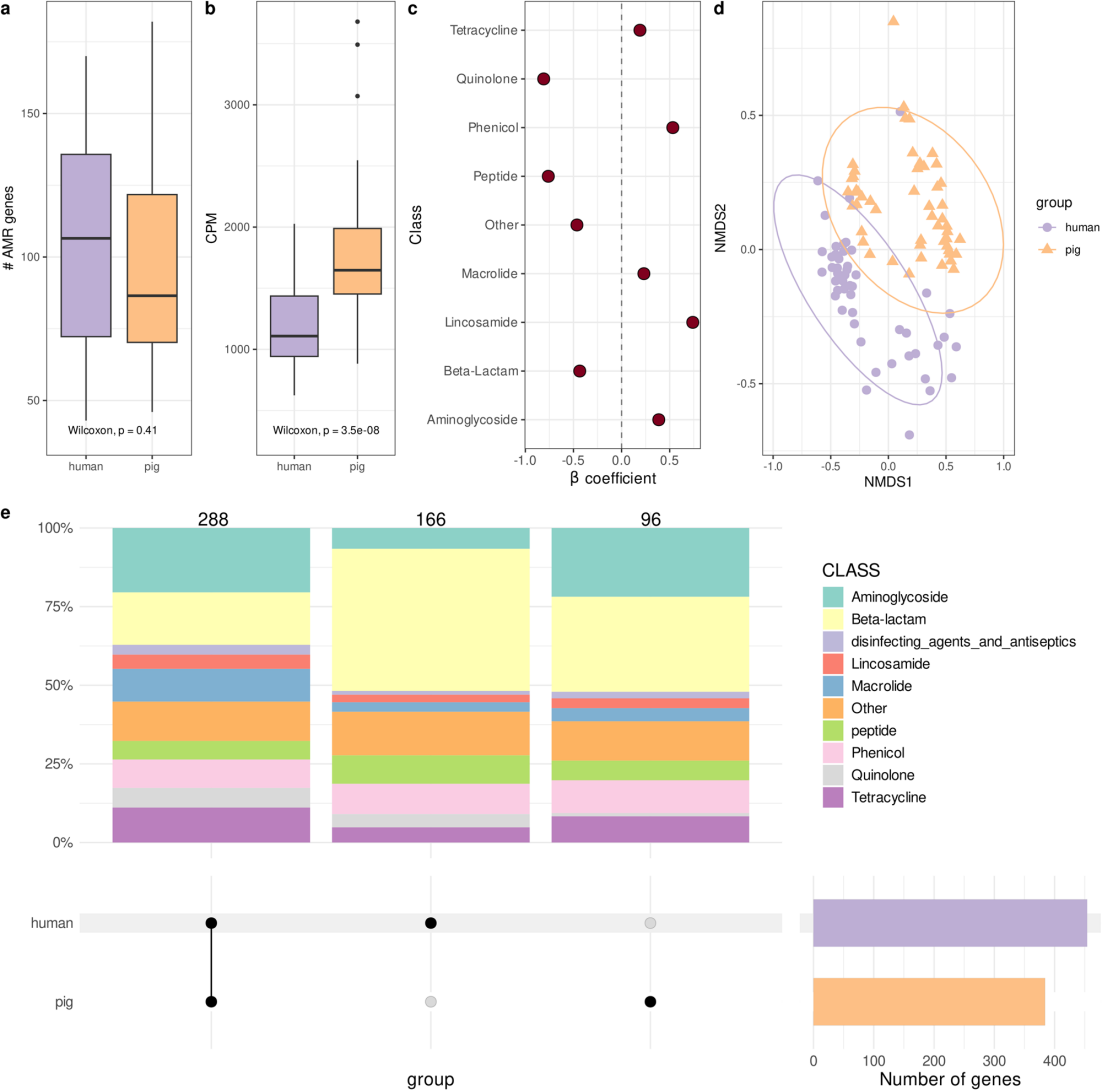
Resistome richness, diversity, and composition. **a** The number of acquired resistance genes. **b** AMR abundance of each gut resistome compared between humans and pigs. **c** Antibiotic classes enriched in the pig gut resistome relative to humans. **d** Non-metric multidimensional scaling (NMDS) analysis based on Bray-Curtis dissimilarities of gut resistome (stress value = 0.10). **e** Upset plot showing shared ARGs. The above panel illustrate ARGs detected by antibiotic class: ARGs shared between hosts (*n* = 288); distinct to humans (*n* = 166); distinct to pigs (*n* = 96). The below panel describes total ARGs carried by humans (*n* = 288 + 166 = 454 ARGs); pigs (*n* = 288 + 96 = 384 ARGs)

Across all samples, 5297 ARGs were identified in livestock workers and 4973 in pigs. Although the number of ARGs was slightly higher in livestock workers, this difference was not statistically significant (*p* = 0.47; Fig. 1a). In contrast, the total AMR load was significantly higher in pigs than in livestock workers (*p* = 3.5e-08; Fig. 1b). Compositionally, resistance determinants against lincosamides, phenicols, aminoglycosides, macrolides and tetracyclines were enriched in pig gut resistomes, whereas livestock workers dominated by genes conferring resistance to β-lactams, quinolones, and peptide antibiotics (Fig. 1c). The resistome clustered significantly between the two hosts, as confirmed by PERMANOVA analysis (*p* < 0.001; Fig. 1d). To reduce the bacterial biomass bias on AMR load, we performed one additional normalisation method (RPKG = reads per kb per genome equivalent) (Supplementary Fig. 2). The total AMR load of pigs remained higher than those of livestock workers (*p* = 0.0014), after correcting to sequenced microbial contents (Supplementary Fig. 2).

### Shared and host-specific ARGs

The extent of ARGs sharing between pigs and livestock workers was further examined. Of the 550 unique ARG types identified, 288 were shared between pigs and livestock workers, while 96 and 166 ARGs were exclusive to pigs and livestock workers respectively (Fig. 1e). The shared resistome was dominated by genes conferring resistance to aminoglycosides (20.4%; e.g. *aad9*, *aadE, aac(6’)-lm, aph(3”)*), followed by β-lactams (16.7%) and tetracyclines (11.1%; e.g., *(tet(O), tet(Q), tet(L*), *tet(W)*, *tet(X), tetA(P)*). Resistance determinants to trimethoprim, nitroimidazole, and sulfonamide (grouped as ‘Other class,’ 12.5%) were also commonly detected in both hosts. Genes within β-lactamase family were prevalent in both pigs and livestock workers, particularly *bla*_ACI-1_, *bla*_EC_, *bla*_OXA-347_, and bla_TEM-_. *bla*_OXA-58_ however, was frequent in pigs but rare in livestock workers. In contrast, *bla*_CTX-_ and *bla*_SHV-_ variants were observed exclusive among livestock workers. Other clinically relevant ARGs that were detected included *optrA* (18/50 pigs; 14/50 livestock workers), *mcr* (6/50 pigs; 4/50 livestock workers), and sporadic vancomycin resistance genes, with the *vanB* cassette identified in one livestock worker and two pig metagenomes, and *vanD* detected only among livestock workers (3/50). Detailed β-lactamase variants distributions as well as other resistant genes are provided in Supplementary Data 1.

### ARGs and mobile genetic elements

**Fig. 2:**
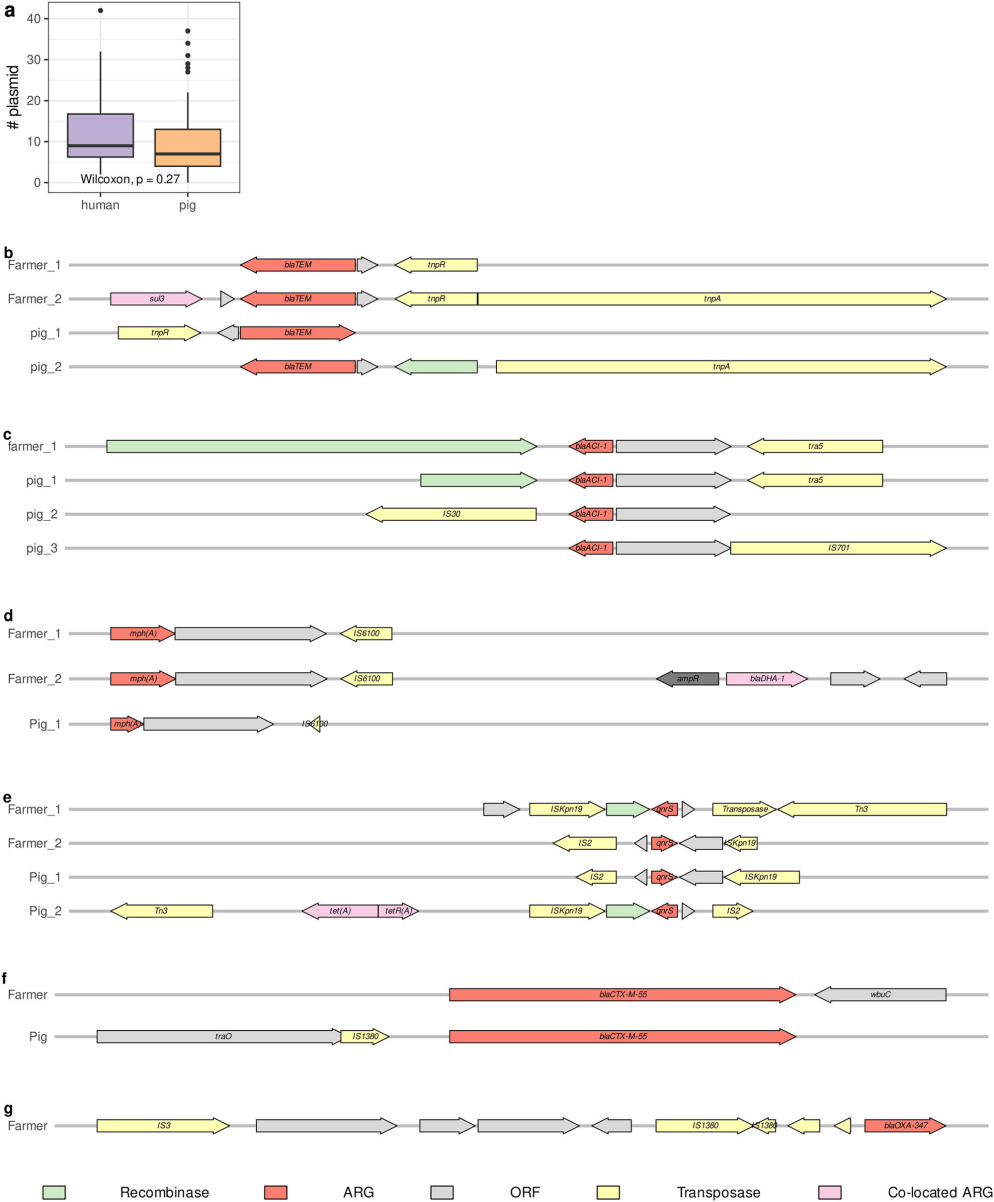
Number of typable plasmids. **a** Gut resistome compared between human and pigs. Aligned contigs between humans and pigs harbouring AMR genes (**b**). *bla*_TEM-1;_ (**c**). *bla*_ACI-1;_ (**d**). *mphA*; (**e**). *qnrS1*; (**f**). *bla*_CTX-M-55;_ (**g**). *bla*_OXA347_.

A greater number of typable plasmids were identified in livestock workers, although the difference was not significant (*p* = 0.27; Fig. 2a). The number of typable plasmids was positively correlated with resistome, indicating that most AMR genes were carried on plasmids (Pearson correlation: R² = 0.79, *p*-value < 0.001). ARGs located on mobile contigs (defined as ARGs co-located with MGEs within 5 kb^4,6^) were examined. Both transposases and insertion sequence (IS) elements were frequently observed in proximity to ARGs. The most common pattern of *bla*_TEM-1_ carrying contigs included the transposon Tn1331, observed in eight livestock workers and four pig samples, while *tnpA* (Tn3 family transposase), was present in two contigs from one livestock worker and one pig (Fig. 2b). The IS3 family transposase *tra5* was the most prevalent element located within 0.8 kb of the *bla*_ACI-1_ in both livestock workers (15/27) and pigs (13/49), while IS30 and IS701 were detected exclusively in pigs (Fig. 2c). Contigs carrying both the macrolide resistance gene *mphA* and the IS6-like transposase *IS6100* were found in both hosts (Fig. 2d). Among livestock workers, the quinolone resistance gene *qnrS1* frequently co-occurred with IS2 and transposase *ISKpn19* (Fig. 2e). Finally, both *bla*_CTX-M-55_ and *bla*_OXA347_ were found in close proximity to IS1380 family transposase IS613 (Fig. 2f, g).

### Microbiota Composition of Pigs and Livestock workers

**Fig. 3:**
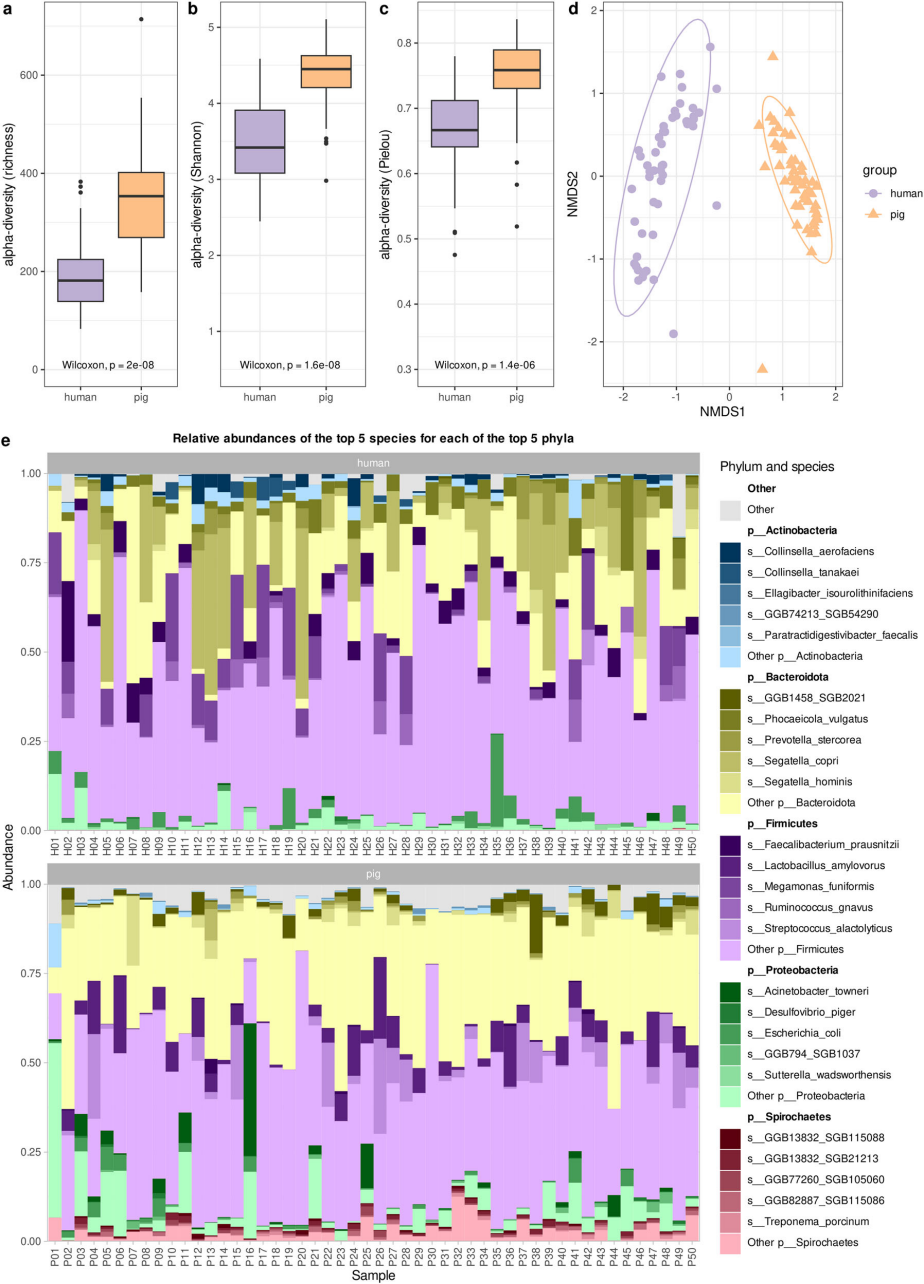
Faecal microbiota profiling at species-level. **a** Alpha diversity richness. **b** Shannon diversity. **c** Pielou evenness. **d** Non-metric multidimensional scaling (NMDS) analysis based on Bray-Curtis dissimilarities (stress value = 0.11). **e** Relative abundance of the five most abundant species corresponding to their respective phyla detected among humans (above) and pigs (below).

In contrast to the resistome, pig microbiotas were significantly richer and more diverse than those of livestock workers. Pigs showed higher number of observed species (*p* = 2 × 10^−8^; Fig. 3a), greater Shannon diversity (*p* = 1.6 × 10^−8^) and higher Pielou evenness (*p* = 1.4 × 10^−8^; Fig. 3b, c). Microbial community structure also markedly differed between hosts by PERMANOVA (*p* < 0.001; Fig. 3d). Across all samples, 2375 species representing 25 phyla were identified, with *Firmicutes* (52.1%) and *Bacteroidetes* (33.9%) dominating in gut microbiota. *Spirochaetes* were observed high among pigs, while *Actinobacteria* in livestock workers (Fig. 3e). Bacteria constituted the majority of the microbiota in both hosts, when only four eukaryotic species at low relative abundance (<0.1%) and 12 archaeal species at variable levels (0–4.6%) were detected.

*Proteobacteria* although less abundant (3.2%), displayed host-specific signatures. Taxa contributions at family and species levels are described in Supplementary Fig. 3. *Moraxellaceace* (represented by *Acinetobacter spp*.) were enriched in pigs (0.16%), relative to livestock workers (*p* = 5.4 × 10^−5^). *E. coli* was detected in nearly all livestock worker samples (48/50, 0.00%–25.8%) and in 43/50 pigs at lower relative abundance (0.00%–6.8%; *p* = 0.014). ESKAPE pathogens observed in the gut microbiota are at low relative abundance (<7%), with *Enterococcus*, *Enterobacter* spp., *Klebsiella pneumoniae* and *Pseudomonas aeruginosa* variably present in livestock workers. *Acinetobacter baumannii* and *Staphylococcus aureus* were absent in both hosts.

### Association between Resistome and Microbiota

**Fig. 4:**
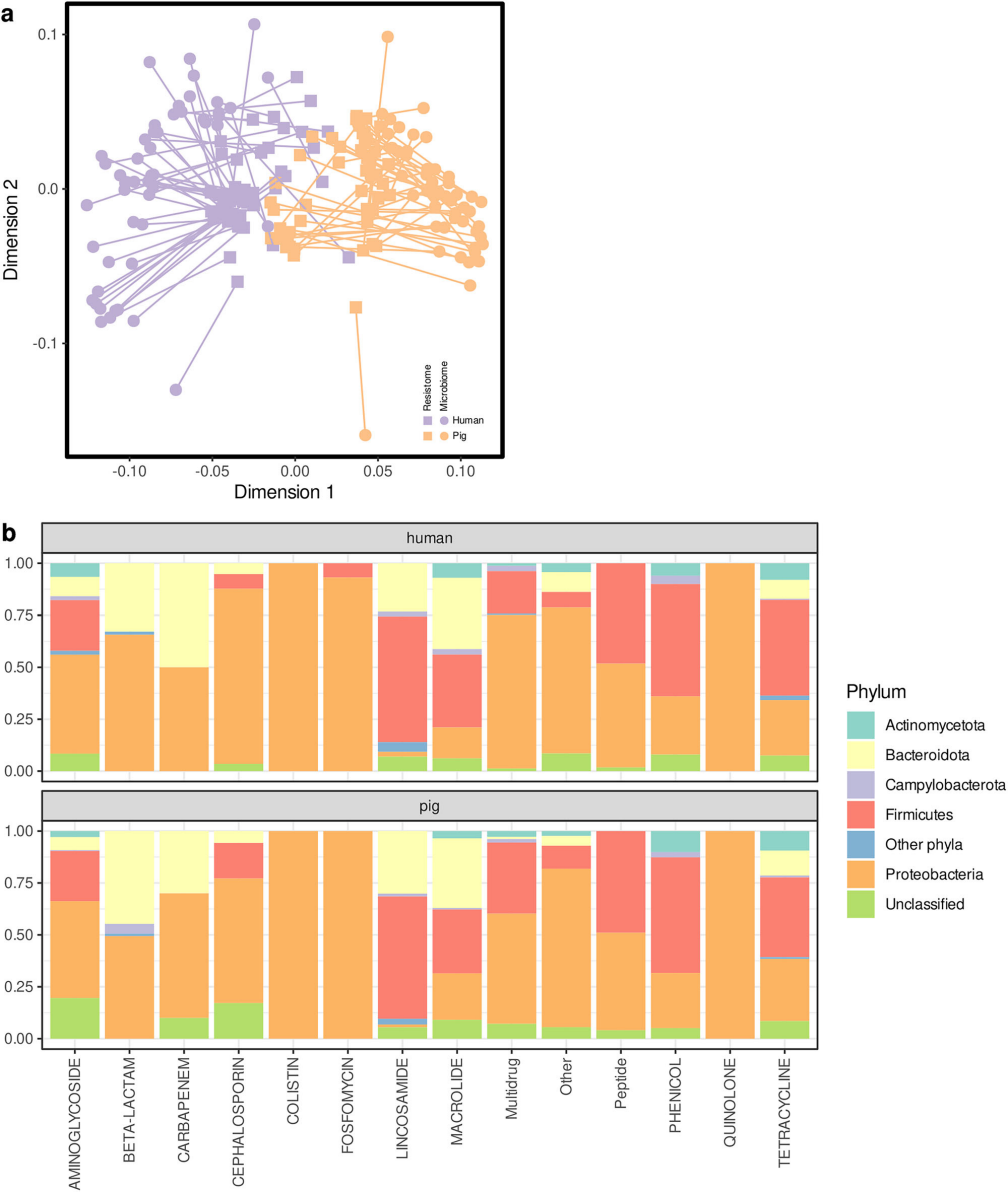
Correlation of Resistome and Microbiota. **a** Procrustes analysis (correlation 0.53, *p* = 0.001, *n* = 100). **b** Stacked bar plot of bacterial ARGs at phylum level stratified by antibiotic class in humans (above) and pig (below).

Procrustes analysis was performed to assess the extent to which the resistome correlated with the bacterial composition of the microbiota. AMR compositions were significantly shaped by the bacteriomes (*p* = 0.001; Fig. 4a). However, the strength of the taxonomy and AMR correlation differed between the hosts: livestock workers exhibited a lower Procrustes correlation (*r* = 0.32, *p* = 0.021) compared to pigs (*r* = 0.60, *p* = 0.001).

Taxonomic assignment of ARG-carrying contigs (ACCs) using Kraken2 showed that most ACCs belonged to *Proteobacteria* (primarily *Gammaproteobacteria*), followed by *Firmicutes* and *Bacteroidetes* (Fig. 4b). Within *Proteobacteria*, members of *Enterobacterales* were the primary carriers of colistin (*mcr*) and quinolone (*oqx, emrA, emrB, emrR, qnr*) resistance genes. *E. coli* (in both hosts) and *Klebsiella* spp. (in livestock workers), also harboured diverse β-lactamase genes. In contrast, *bla*_OXA-347_ was mostly observed in *Bacteroidales*, alongside *cfx* and *cfiA*, whereas *bla*_ACI-1_ was primarily associated with *Negativicutes*. Among lincosamide genes, *lnuA/B/G* were mainly found in *Bacilli*, whereas *lnuAN2* and *lnuF* was carried predominantly by *Bacteroidota* and *Gammaproteobacteria*. *Firmicutes* also carried tetracycline, peptide, and phenicol resistance genes, while macrolide resistance genes were distributed across *Bacteroidota*, *Firmicutes*, and *Gammaproteobacteria*. *Actinomycetota* additionally carried *cmx*, *tetZ*, *tetW*, and *ermX*.

Correlation analyses further supported likely bacteria hosts of ARGs. For example, although contigs carrying *bla*_OXA-58_ could not be taxonomically classified due to short length, their abundance strongly correlated with *Acinetobacter towneri* and *A. amyesii* (*ρ* = 0.8, *p* < 0.001), suggesting these species as potential carriers of this carbapenemase. Similar co-occurrence patterns were observed for *bla*_OXA-164_ and *bla*_CARB-11_. In pigs, *A. towneri* and *A. amyesii* were additionally associated with the abundance of several ARGs including *sul2*, floR, *ant(3”)*, *aph(6)-Id*, *aac(3)* (aminoglycosides); *mphB* and *mphE* (macrolides); *tet(39)* and *tet(Y)* (tetracycline efflux pump) (Suppl. Fig. 4; Supplementary Data 2). In contrast, *Klebsiella* formed the major ARGs-taxa correlation in livestock workers, co-occurring with *oqx* gene family (fluoroquinolone), *bla*_SHV_ and *bla*_ACT_ (SHV and AmpC β -lactamases) and *fosA* (fosfomycin) (Suppl. Fig. 5; Suppl. Data 3).

### Microbial exchange at the strain level

StrainPhlAn analysis identified 417 species-level genome bins (SGBs) with sufficient markers across at least four samples. Of these, 137 were exclusive to livestock workers, 186 to pigs, and 94 SGBs presented in both hosts (Supplementary Data 4). *E.coli* (SGB10068) was the most common SGB, detected in 35 livestock workers and 15 pigs, followed by *Segatella copri* (SGB1626; 34 livestock workers, 11 pigs).

**Fig. 5:**
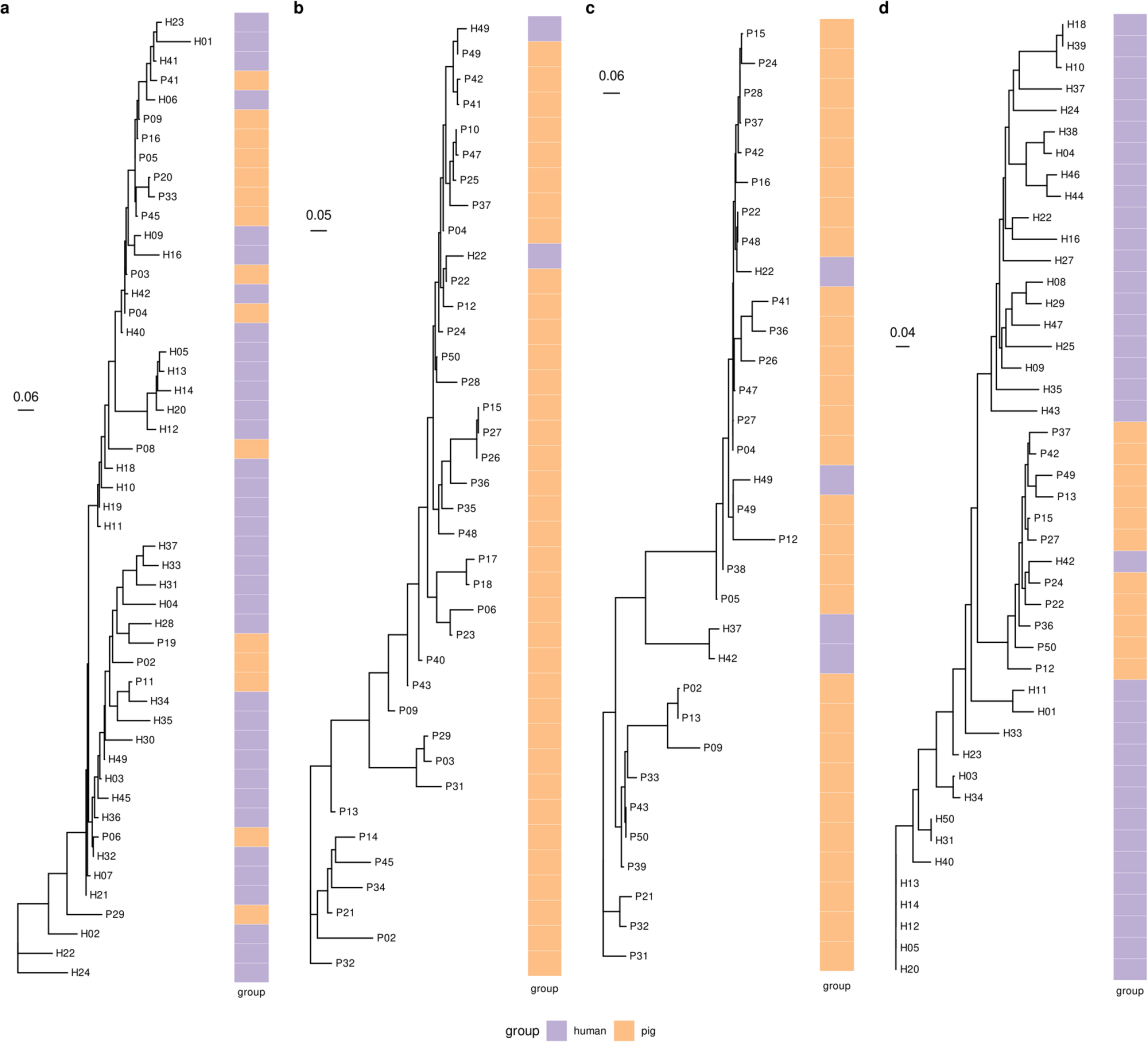
Phylogenetic analysis revealed intermixed clusters between hosts, indicating strains sharing events at the human-animal interface. **a** *Escherichia coli*. **b** *Lactobacillus amylovorus***. c** *Streptococcus alactolyticus*. **d** *Segatella copri.*

Phylogenetic analysis of *E. coli* revealed intermixed clusters between hosts, indicating strains sharing events at the human-animal interface (Fig. 5a). Several strains within metagenome of pigs and livestock workers showed minimal genetic distances. For example, the H19 strain clustered closely with pig strains P03, P04, P09, and P16 (1–4 mutations across ~70,000 sites). Similarly, the P05 strain was genetically closer to multiple livestock workers (H19, H32, H36, H40; 0–6 mutations / ~ 60,000–70,000 sites) than to its paired livestock worker H05 (82 mutations/ 62728 sites). Comparable patterns were observed in other farms, with intra-farm pig–human pairs (e.g., Farm 41, Farm 09, Farm 16) showing related strains but with less impact than cross-farm mixing. High-resolution analysis of *E. coli* MAGs recovering from metagenomic data confirmed these findings (Supplementary Fig. 6). High ANI between draft *E.coli* genomes (mean 99.3% ± 0.1%) of human–pig pairs such as H41–P41, H09–P09, and H09–P16 were observed (Supplementary Fig. 7).

Beyond *E. coli*, strain-level sharing was also observed for *Lactobacillus amylovorus* and *Streptococcus alactolyticus*, despite their predominance in pigs. Notably, closely related strains were identified across livestock workers and pigs in Farm 22 and Farm 49 (Fig. 5b, c). In contrast, *Segatella copri* formed host-specific clusters, with the exception of one livestock worker strain grouping with pig isolates (Fig. 5d). Together, these results indicate strain-level relatedness and possible sharing of several species at the human–animal interface.

## Discussion

In this study, shotgun metagenomic sequencing was applied to characterise the abundance, diversity, and exchange of the ARG’s and microbiota between humans and pigs in a Vietnamese farm setting. The findings revealed that livestock workers harboured a richer and more diverse resistome, although AMR load was higher in pigs. The gut resistome was distinctly shaped by host-specific microbiota and antibiotic selection pressures. At a finer resolution, strain-level analysis revealed close relatedness of strains detected in pigs and livestock workers, consistent with possible sharing at the human–animal interface. The similarities patterns ARGs, of which *E. coli* and other *Gammaproteobacteria* were the primary carriers, were shared between two hosts. Moreover, the frequent occurrence of ARGs with plasmids and MGEs suggests a potential of horizontal gene transfer across the human-animal interface, underscoring the close linkage of AMR ecology across hosts within a One Health context.

A marked difference was observed in the gut microbiota and resistome profiles of pigs and livestock workers. Such differences are expected in this cohort, as gut microbiota composition is shaped by multiple host-associated factors^16,17^, including diet, physiology, living environment, and antimicrobial exposure, all of which differed between pigs and livestock workers. Nevertheless, the gut microbiota of livestock workers retained distinct features commonly reported in rural Vietnamese populations and in individuals with intensive pig exposure, including enrichment of *Prevotellaceae*^18,19^. This family is widely regarded as a hallmark of microbiota associated with grain- and vegetable-rich diets common in many LMIC populations^17^ and has also been reported to be more prominent in pig workers than in individuals exposed to cattle^19^, poultry, or in non-exposed controls^20^.

Likewise, the resistome of pigs and livestock workers was clearly distinct. These differences were likely shaped by variation in bacterial community structure^21,22^, dominant host-associated taxa, and differing antimicrobial exposure patterns^16^. The taxa–AMR association was observed to be weaker in livestock workers, together with the higher number of typable plasmids, may indicate a greater contribution of mobile genetic elements to resistome structure in humans. Pig resistomes were dominated by ARGs conferring resistance to lincosamides, phenicols, aminoglycosides, macrolides, and tetracyclines, whereas livestock-worker resistomes were relatively enriched in β-lactam, quinolone, and peptide classes. These host-specific resistome patterns are broadly consistent with known differences in antimicrobial exposure between human and livestock settings reported in the literature^23,24^. However, because farm-level antimicrobial-use data in this study were based primarily on questionnaire responses and were not quantified in detail by drug class, dose, or duration, these associations should be interpreted cautiously and not as direct evidence of selection pressure within individual farms.

The observation that pigs carried a higher total AMR abundance, whereas livestock workers harboured a more diverse resistome, suggests that abundance and diversity capture distinct dimensions of resistome structure. One possible explanation is that repeated selection for a narrower set of ARGs in pigs may increase overall ARG abundance, whereas more heterogeneous microbial and exposure histories in humans may support greater ARG diversity. However, these mechanisms were not directly tested here. In addition, although higher AMR load in pig remained consistent across normalisation to sequencing depth, gene length (CMP/RPKM) and genome-equivalent bacterial biomass (RPKG), absolute measurement was not preformed. Accordingly, this finding should be interpreted as a robust descriptive pattern rather than a fully resolved mechanistic conclusion.

Resistome and microbiota comparisons at higher resolution revealed resemblances between pigs and livestock workers. Approximately 75% (288/384) of ARGs detected in pigs were also observed in livestock workers, including those sharing similar genetic contexts such as co-located transposon elements. This result indicated substantial overlap in ARG and genetic context between hosts, consistent with possible sharing or exposure to common reservoirs. Shared ARGs were dominated by aminoglycoside resistance determinants. Genes mediating aminoglycoside modification through acetylation (*aac(6’)-lm*), adenylation *(aad9*, *aadE*), or phosphorylation (*aph(3”)*) were ubiquitous in both hosts and associated with *Enterobacteriaceae* (e.g. *aph(6)-Ic*, *aph(3”)*) or *Enterococcaceae (aad9)*. The prominence of aminoglycoside resistance likely reflects the extensive use of this antibiotic class in pig farming^25^. Notably*, rmtF*, conferring high-level aminoglycoside resistance^26^, was restricted in pigs, whereas lower-level determinants were shared across hosts, indicating a stronger selection pressure in pigs. Overlapping ARGs between pigs and respective livestock workers also included genes conferring resistance to tetracyclines, to a lesser extent to β-lactams (*bla*_OXA-347_, *bla*_ACI_, *bla*_TEM_), and phenicols (*floR*, *cat*), reflecting the common veterinary use of chlortetracycline and florfenicol. The detection of *optrA* despite the limited veterinary access to linezolid further supports the co-selection via phenicol use, as *optrA* frequently acquired with *fexA* (phenicol resistance) and/or *ermA* (macrolide resistance), both prevalent in pig samples in this study^27^. Although such resistance determinants may also occur naturally within healthy gut resistomes^28^ or emerge from unregulated antibiotic use in Vietnam^11^, the antibiotics routinely administered in pig farming remain key drivers of resistome similarity between pigs and livestock workers.

Phylogenetic analysis revealed closely related *E.coli* strains in both pigs and livestock workers metagenomes (0–6 SNPs per approximately 70,000 sites of markers genes). Most *E.coli* strains belonged to Clermon Typing phylogroups A and B1, typically representing commensal strains in humans and livestock^29^. Previous studies have reported clonal dissemination between farmers and livestock using multilocus sequence typing^30^, whole genome sequencing^31^ or metagenomics^6^, though such strain-sharing events generally occurs sporadically across limited farm pairs. In this study, *E. coli* MAGs shared between livestock workers and pigs were not confined to single farms. The close proximity of the sampled sub-villages, together with animal trade, shared environments, and frequent social interactions, may explain the observed strain relatedness across hosts and farms. The absence of environmental sampling, including soil, water, feed, manure, and farm equipment, limited our ability to evaluate common-source reservoirs and indirect transmission pathways. Likewise, the cross-sectional design prevented assessment of temporal persistence and transmission directionality. StrainPhlAn primarily captures the dominant strain, which may lead to underrepresentation of co-existing minor variants within mixed-strain communities^32^. In addition, *E. coli* populations are subject to continuous microevolution within individual hosts^33^, while the pooling of pig faecal samples could further enhance within-sample heterogeneity. Longitudinal sampling, environmental metagenomics, and cultured isolates sequencing will be valuable to further understand *E. coli* transmission dynamics. Nonetheless, the results highlight *E. coli* as an important commensal associated with shared ARG patterns across humans and pigs.

In this study, the *bla*_OXA-58_ was associated with non*-baumannii Acinetobacter* species, a species predominant in pigs gut microbiota. The acquisition of carbapenem-hydrolysing class D β-lactamases (e.g., OXA-23, OXA-24 or OXA-58) has contributed to the global spread of carbapenem-resistant *Acinetobacter baumannii* and non*-baumannii* isolates across clinical^34^, animal^35^ and environmental resources^36^. The detection of OXA-58 in pigs likely reflects environmental dissemination^36^. This gene is located on plasmids co-harbouring other resistance determinants such as the *bla*_NDM−1_ and *tet*^35,37^, suggesting co-selection under continued tetracycline in veterinary use. These findings warrant further investigations on carbapenem-resistant *Acinetobacter spp*. from non-clinical isolates. In contrast, *bla*_NDM_ were rare, detected in only one livestock worker (*bla*_NDM-5_) and one pig (*bla*_NDM-1_), consistent with previous reports showing low community prevalence in Vietnam (4/93 human, 2/45 animal samples)^13^. Pigs are unlikely to be the primary reservoir for this metallo-beta-lactamase, likely because carbapenem selective pressure is not directly applied to pig populations. Although, *bla*_NDM-1_ is widespread among *Klebsiella* and *Pseudomonas* clinical isolates in Vietnam, colonisation by these species was not detected in the pig microbiota examined in this study. *E. coli* is also known as carrier of *bla*_NDM_^38^, though their common host source could be chicken and poultry rather than pig population.

The prevalence of plasmid-mediated colistin resistance genes (*mcr-1*, *mcr-3* and *mcr-10)* 12% in pigs and 8% in livestock workers was relatively low, compared to earlier community-based studies within Vietnam, reporting *mcr-1* prevalence from 36.6% to 88% in human faeces and 18.9–94.4% in animals^13,39^. The relatively low prevalence of plasmid-mediated *mcr* genes in this dataset contrasts with earlier reports from Vietnam. However, because phenotypic colistin susceptibility testing was not performed, the clinical significance of these findings remains uncertain. We therefore cannot determine whether low *mcr* detection reflects reduced colistin resistance overall, alternative chromosomal mechanisms, differences in study populations, or temporal changes in antimicrobial selection pressures.

This study has several limitations. The first limitation of this study is the absence of culture-based phenotypic antimicrobial susceptibility testing. Consequently, the detection of ARGs, including clinically important determinants such as *bla*_NDM_, *bla*_OXA-58_, *mcr*, and *optr*A, should be interpreted as evidence of genetic resistance potential rather than confirmed functional resistance in viable or dominant bacterial populations. Second, the cross-sectional design precludes inference of temporal sequence, directionality, or causality of strain or ARG sharing between pigs and livestock workers. Third, because environmental samples were not collected, we could not assess the contribution of shared farm environments, water, feed, manure, household surroundings, or broader community reservoirs as alternative sources of the observed overlap. Fourth, the absence of a non-exposed control group limited our ability to determine the extent to which occupational pig exposure specifically shaped the microbiota and resistome profiles. Finally, antimicrobial-use data were based primarily on questionnaires and limited regional evidence, rather than direct longitudinal usage measurements.

From a policy perspective, more targeted antimicrobial stewardship in Vietnamese smallholder pig farming is supported by these findings. In particular, the predominance of ARGs linked to tetracyclines, aminoglycosides, lincosamides, phenicols, and macrolides in pigs suggests that these antibiotic classes should be prioritized for stewardship, monitoring, and reduction where routine or prophylactic use persists. Enhanced oversight of premixed and medicated feeds, more precise labelling, and more stringent enforcement of existing regulations on veterinary antimicrobial access may help curtail selection pressure at farm level.

In conclusion, the findings highlight the intricate and interconnected relationships between farm animals and livestock workers. Although the principal determinant shaping both the microbiota and resistome are largely host-specific, notable cross-species linkages were evident. Antibiotic use in pig farming likely contributes to resistome enrichment in pigs and may shape overlapping ARG patterns observed at the human–pig interface. The data underscore the role of *Gammaproteobacteria* as important carriers of clinically relevant ARGs and support the possibility of shared reservoirs or strain sharing, while direct transmission pathways remain unresolved in the present study. Taken together, our findings support substantial overlap and close genomic relatedness across hosts, but they should not be interpreted as proof of direct pig-to-human or human-to-pig transmission.

## Methods

### Ethical approval

The study was approved by the Institutional Review Board of the 108 Military Central Hospital, Hanoi, Vietnam (108MCH/RES/I-CRECT-V1.1-D2-06-04-2021).

### Study design and sample collection

Fifty small-scale pig farms (<40 pigs per farm) in Northern Vietnam were enrolled in July 2022. Livestock workers were largely 90% male, median age 48 years [IQR: 43.5–57], with a farming experience 4–27 years. Most farms raised piglets and slaughter pigs, with limited sow rearing. Semi-commercial feed was used in 47/50 farms (94%), while three relying on fully commercial feed. All pigs were housed in cement-floored cages and vaccinated against classical swine fever, *E. coli*, salmonellosis, pneumonic pasteurellosis, porcine reproductive and respiratory syndrome (PRRS), and foot-and-mouth disease (FMD). Detail demographics are provided in Supplementary Data 5. From each farm, paired faecal samples were collected from pigs and the corresponding livestock caretaker of that farm. Pig samples were obtained by pooling fresh droppings from 3–5 cages, homogenising, and swabbing with sterile cotton applicators. Equally, livestock caretaker self-collected faecal swabs immediately after defecation. All swabs were preserved in DNA/RNA Shield (Zymo Research GmbH, Freiburg im Breisgau, Germany) and transported at 4 °C to the laboratory within 12 h .

### DNA extraction and Shotgun metagenomic sequencing

Genomic DNA was extracted using the ZymoBIOMICS™ DNA Miniprep Kit (Zymo Research GmbH, Freiburg im Breisgau, Germany) following the manufacturer’s protocol, with a modified lysis step: 250 µl of sample and 750 µl of lysis buffer were processed in ZR BashingBead™ tubes on a PowerLyzer 24 Homogenizer (Qiagen, Hilden, Germany) at 4000 rpm for 1 min × 3 cycles. DNA was eluted in DNase/RNase Free water and stored at − 20 °C. To control for contamination, the ZymoBIOMICS Microbial Community Standard was included as a positive control, and a non-template control was extracted and sequenced alongside the samples.

Metagenomic libraries were prepared using the Illumina DNA Prep kit (Illumina Inc., San Diego, CA, USA), pooled and sequenced on the Illumina NextSeq 2000 platform with P4 XLEAP-SBS™ chemistry (paired-end, 2 × 100 bp). The target yield was approx. 20 million paired-end reads per sample, and samples below this threshold yield were sequenced again under identical conditions.

### Species and strain level profiling

Raw reads were quality filtered using fastp (v0.23.2; -q = 30 and -l = 45 parameters)^40^. Host sequences were removed by KneadData (v.0.12.0; default parameters with the –bypass-trim option; https://github.com/biobakery/kneaddata). The human (GRCh38 GCF_000001405.40) and pig genome (GCF_000003025.6) were used as reference genomes to remove host sequences. The resulting reads were processed through MetaPhlAn (v4.1.0)^41^ with the vJun23_202403 database for species-level profiling. Strain-level sharing between pigs and humans was further assessed using StrainPhlAn (v4.1.4)^42^.

### Reconstruction of metagenome assembled genomes (MAGs)

MAGs were reconstructed using MetaWRAP (v.1.3)^43^ yielding 1772 bins (858 high quality, defined as ≥ 90% completeness, ≤ 5% contamination; 914 medium quality, defined as ≥ 50% completeness, ≤ 10% contamination bins). Species identification of MAGs was performed using mash (sub-command screen) by screening each draft genome against a representative genome database composed of each species present in the NCBI Microbial Genomes resource (https://www.ncbi.nlm.nih.gov/genome/microbes/). Full MAG details are presented in Supplementary Data 6.

For *E. coli*, the most abundant clade in both hosts, MAGs were aligned to the reference genome (NC_000913.3) using SKA (Split Kmer Analysis), and this alignment was refined with Gubbins (v3.3.1)^44^ to remove recombination. A total of 101,697 polymorphic sites were retained for the reconstruction of the phylogenetic tree by RAxML (v.8.2.12)^45^. Average nucleotide identity (ANI) was calculated using an all-vs-all comparison of the genomes using ANIclustermap (v.1.3.0; https://github.com/moshi4/ANIclustermap). Resulting phylogenies were visualised in R with the ggtree package^46^. *Escherichia* strains were phylotyped in silico using ClermonTyping^47^.

### Resistome profiling

Resistomes were reconstructed as previously described^48^. Curated reads were de novo assembled using SPAdes (v4.0.0)^49^ with the -meta and -only-assembler options. Acquired ARGs and plasmids were identified by screening draft assemblies against NCBI, CARD, ARG-ANNOT, ResFinder, MEGARes, and PlasmidFinder databases^50–54^ using Abricate (https://github.com/tseemann/abricate) with thresholds of ≥ 90% identity and ≥ 25% coverage. To further examine mobile ARGs content across hosts, ARGs-carrying contigs ( > 500 bp) were extracted and filtered using seqtk (https://github.com/lh3/seqtk), followed by annotation using Bakta (v.10.4)^55^. For abundance estimation, raw FASTQ reads were mapped to identify ARGs using Bowtie2 -very sensitive parameter^56^. Raw mapping counts were normalised for gene length and sequencing depth to generate CPM and RPKM values.

### Statistical analysis

The microbiota and resistome diversity metrics were calculated and visualised using Phyloseq, Complexheatmap, ComplexUpsetR, fantaxtic and ggplot2 in R 4.4.2. The association of host type with beta diversity was assessed using permutational multivariate analysis of variance (PERMANOVA) (method = “bray,” permutations = 999) in the R package vegan. The correlation between bacterial and resistome profile was evaluated using Procrustes analysis (permutations = 9999). The taxon’s relative abundance and abundance of ARGs were tested for correlation using Spearman correlation analysis. Network graphs were constructed with Gephi (v0.9.6).

## Supplementary information

Supplementary Information_R1

Supplementary Data_R1.

## Data Availability

The metagenomic sequencing data (host-decontaminated fastq files) of this study are available in the NCBI/ENA under the BioProject Accession PRJEB90858 (https://www.ebi.ac.uk/ena/browser/view/PRJEB90858). The generated sequence data can be accessed from the ENA or NCBI databases using respective IDs. SRA Experiments ID: ERR15143211- ERR15143258; ERR15143626-ERR15143627; ERR15156790- ERR15156838; ERR15156851-ERR15156852.
