# Supplementary material for: Metagenomic profiling reveals shared resistome signatures between humans and pigs in Vietnamese smallholder farms
Source: NPJ Antimicrob Resist. 2026 Jun 2;4:43. doi: 10.1038/s44259-026-00223-6 (PMC13273033; doi:10.1038/s44259-026-00223-6)
Supplement: Supplementary file 1 — Supplementary Information_R1 [file 44259_2026_223_MOESM1_ESM.pdf]

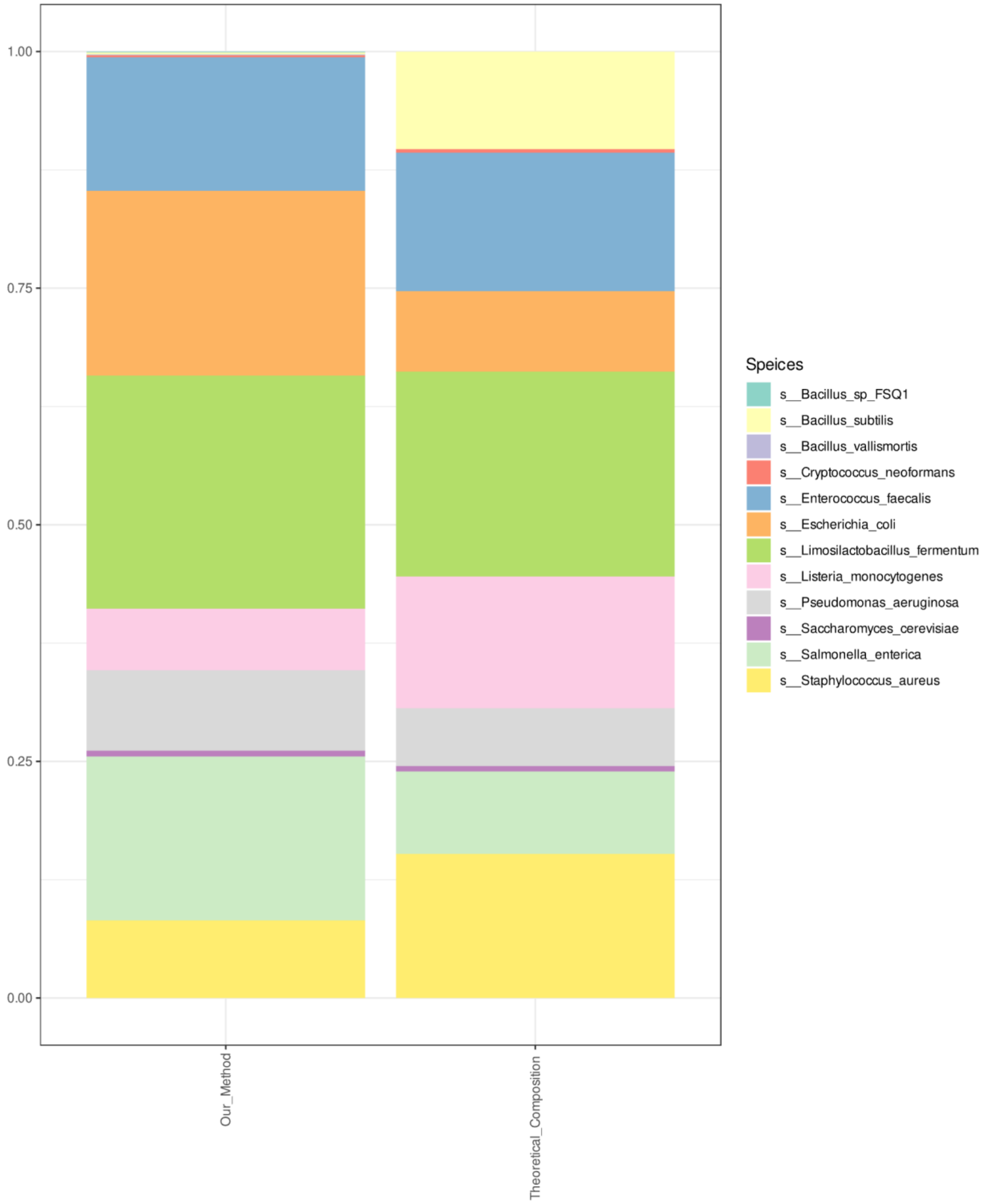

Supplementary Figure 1. Observed species and their relative abundance in positive control: study composition (left) and theoretical composition (right)

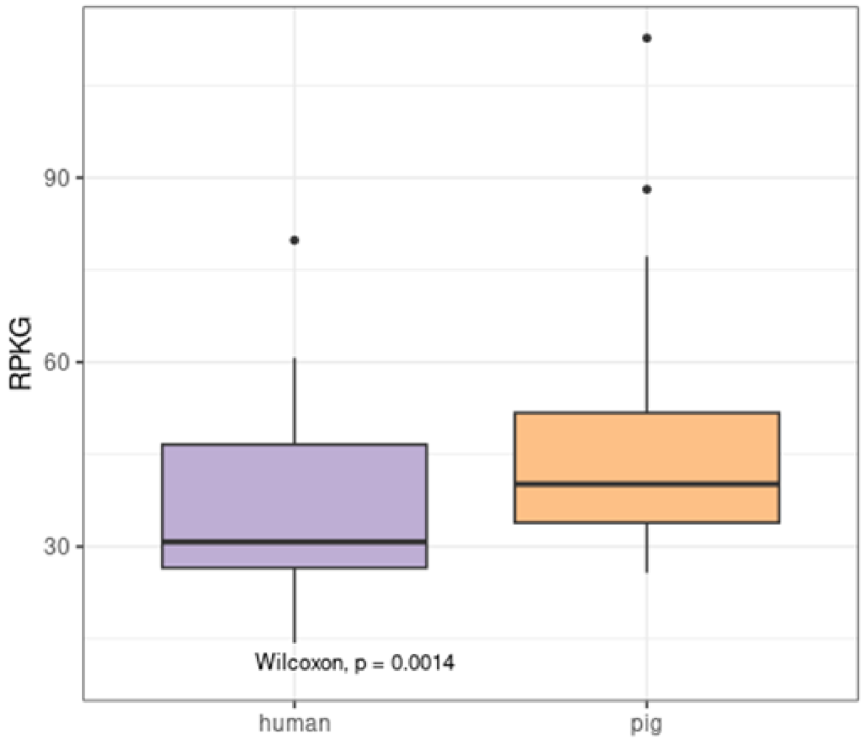

Supplementary Figure 2. Total AMR load represented by reads per kb per genome equivalent (RPKG) of livestock workers and pigs. Genome equivalent value, which estimates the total coverage of microbial genomes present in a sample, is calculated by dividing total bp sequenced to average genome size (AGS) from universal single-copy genes of cellular microbes [1].

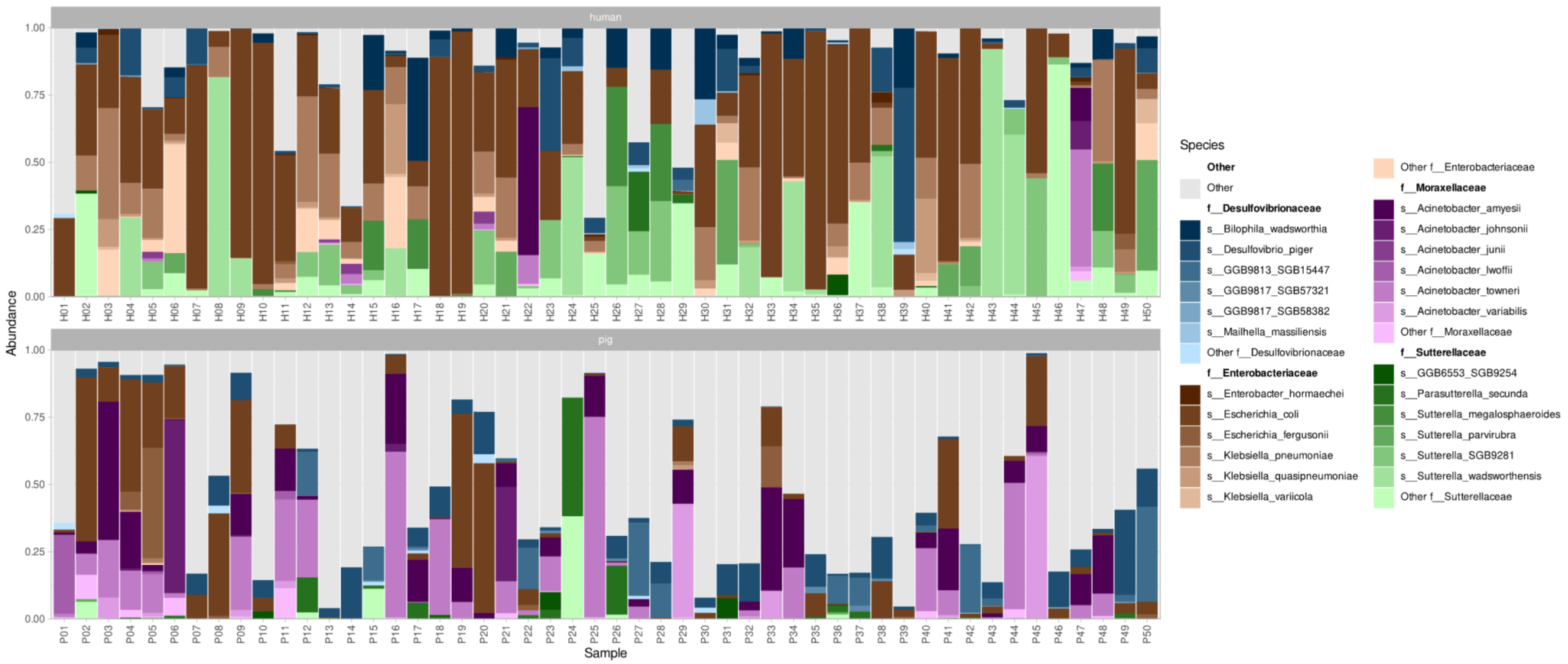

Supplementary Figure 3. Relative abundance of top six species corresponding to their respective family within *Proteobacteria* phylum in humans (above) and pigs (below).

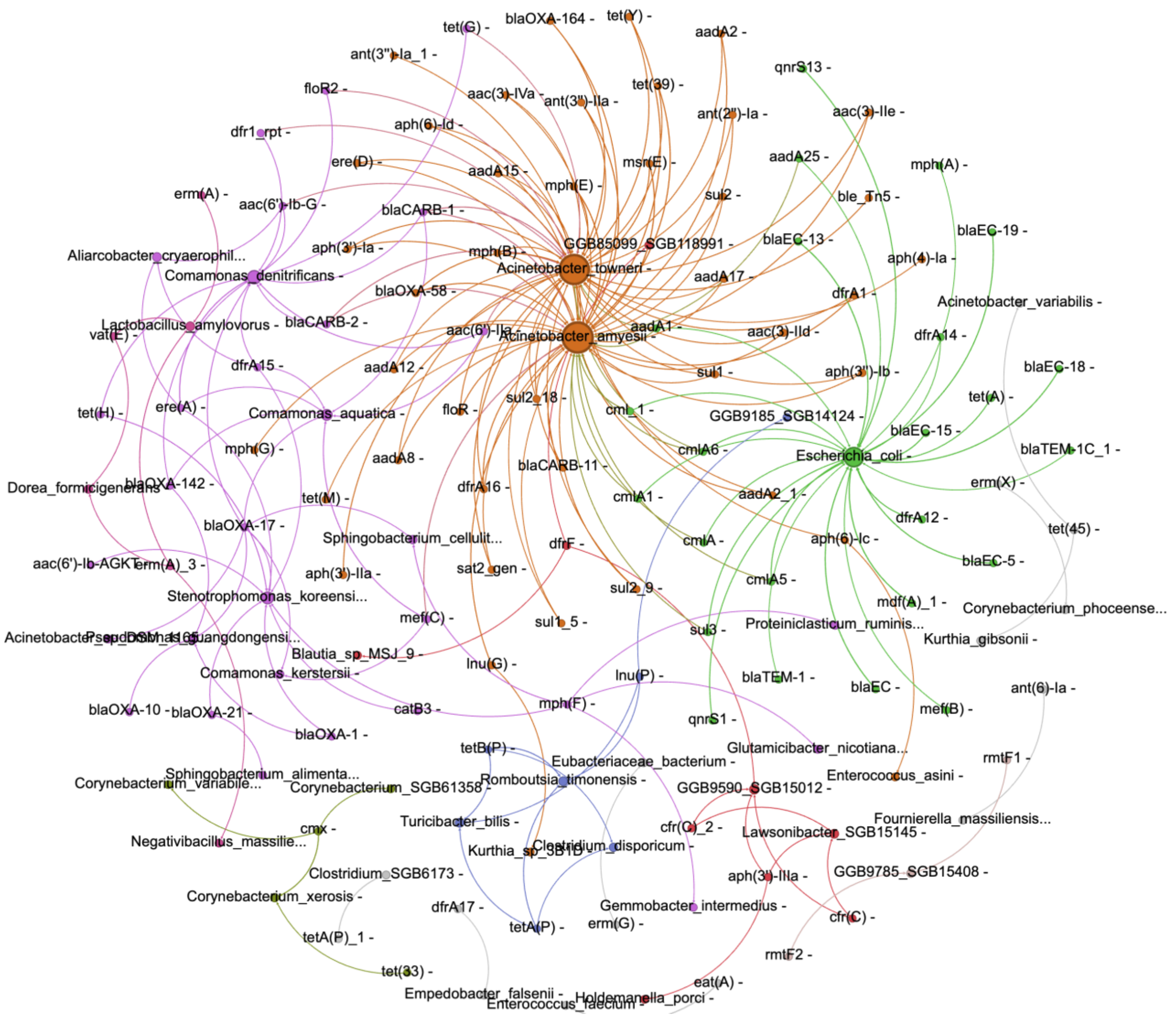

Supplementary Figure 4. Network analysis on the co-occurrence of microbial taxa and ARGs among pigs. Nodes are colored according to community detected by algorithm described here [2]. The size of each node is proportional to its number of connections. An egde represents a strong (Rho ≥ 0.7) and significant (p value < 0.05) correlation.

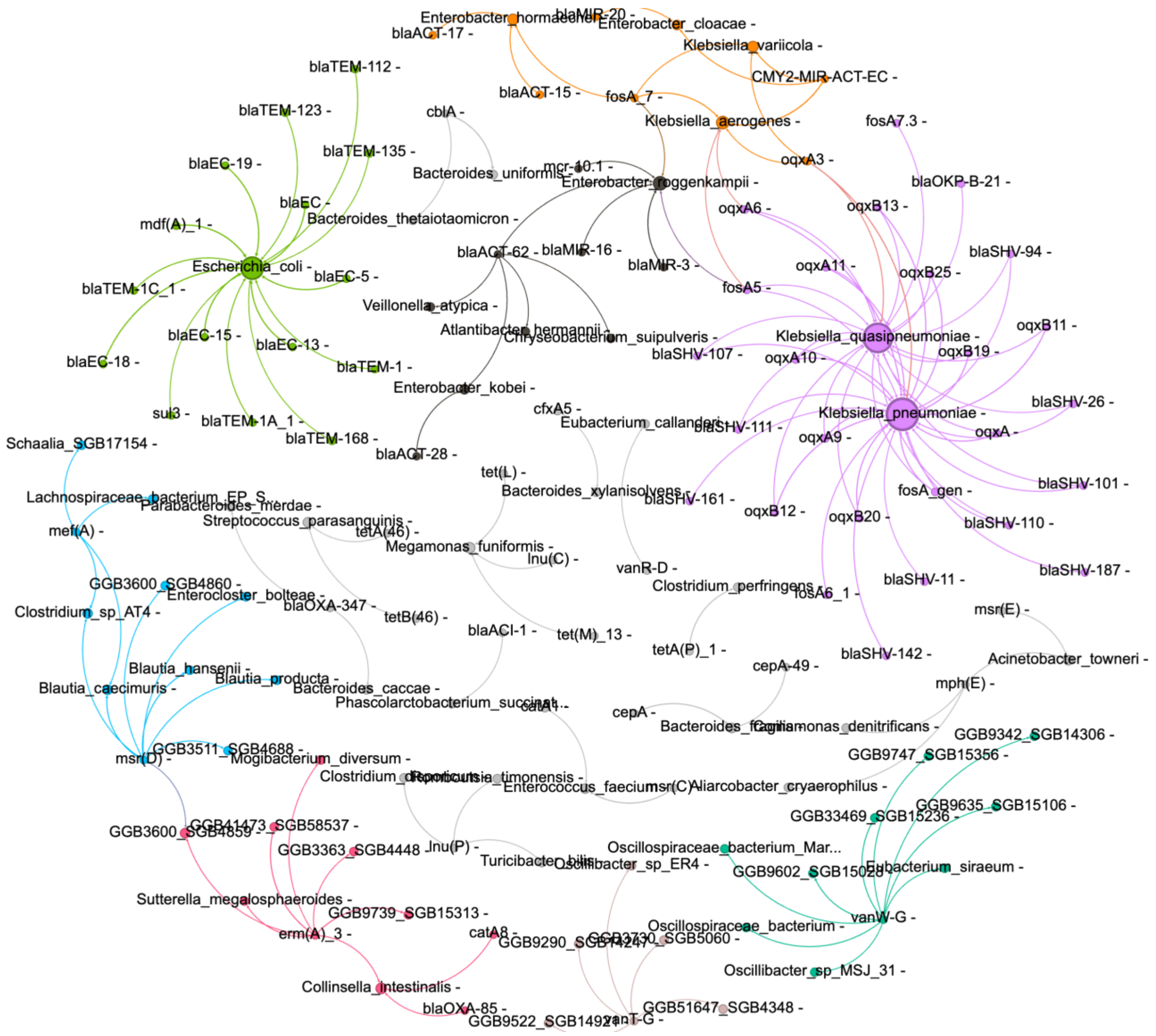

Supplementary Figure 5. Network analysis on the co-occurrence of microbial taxa and ARGs among livestock workers. Nodes are colored according to community detected by algorithm described by [2]. The size of each node is proportional to its number of connections. An egde represents a strong (Rho ≥ 0.7) and significant (p value < 0.05) correlation.

a.

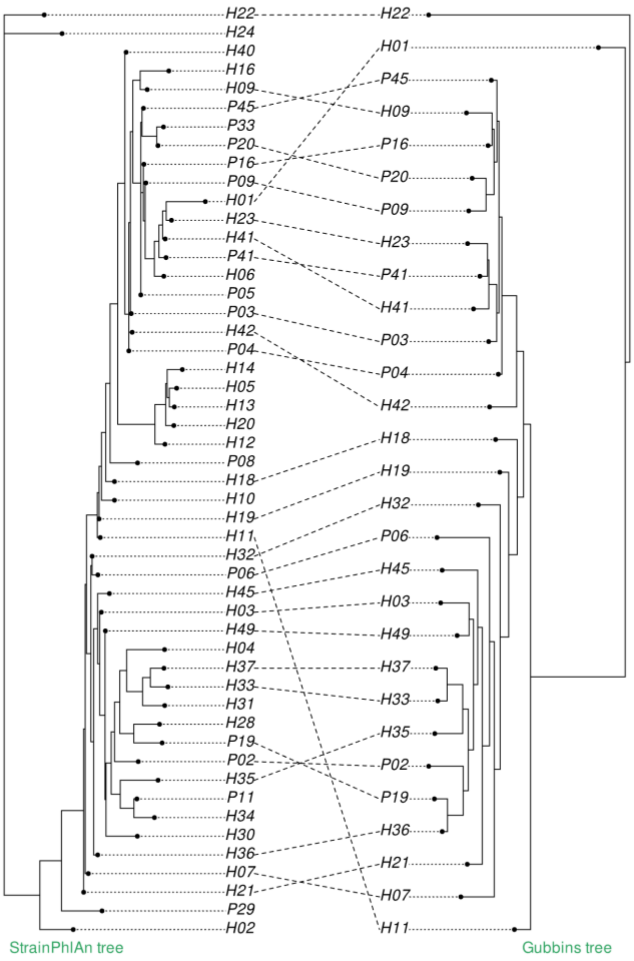

b.

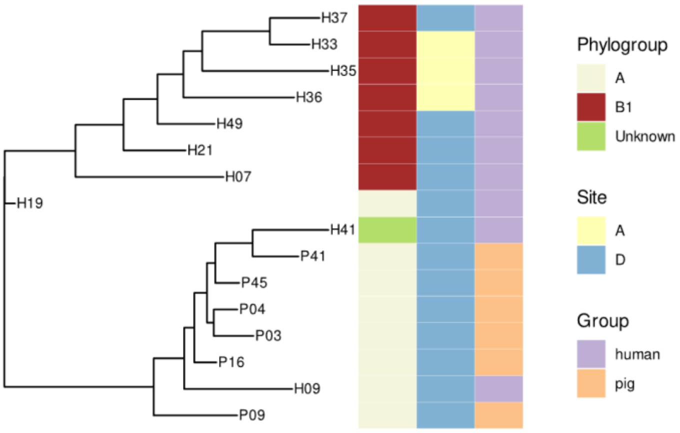

Supplementary Figure 6. (a). Phylogenetic analysis of *E.coli* strain sharing events based on marker genes (StrainphlAn) and putative point mutations outside recombination regions (Gubbins) (**b).** Phylogenetic analysis of *E.coli* MAGs with metadata annotation.

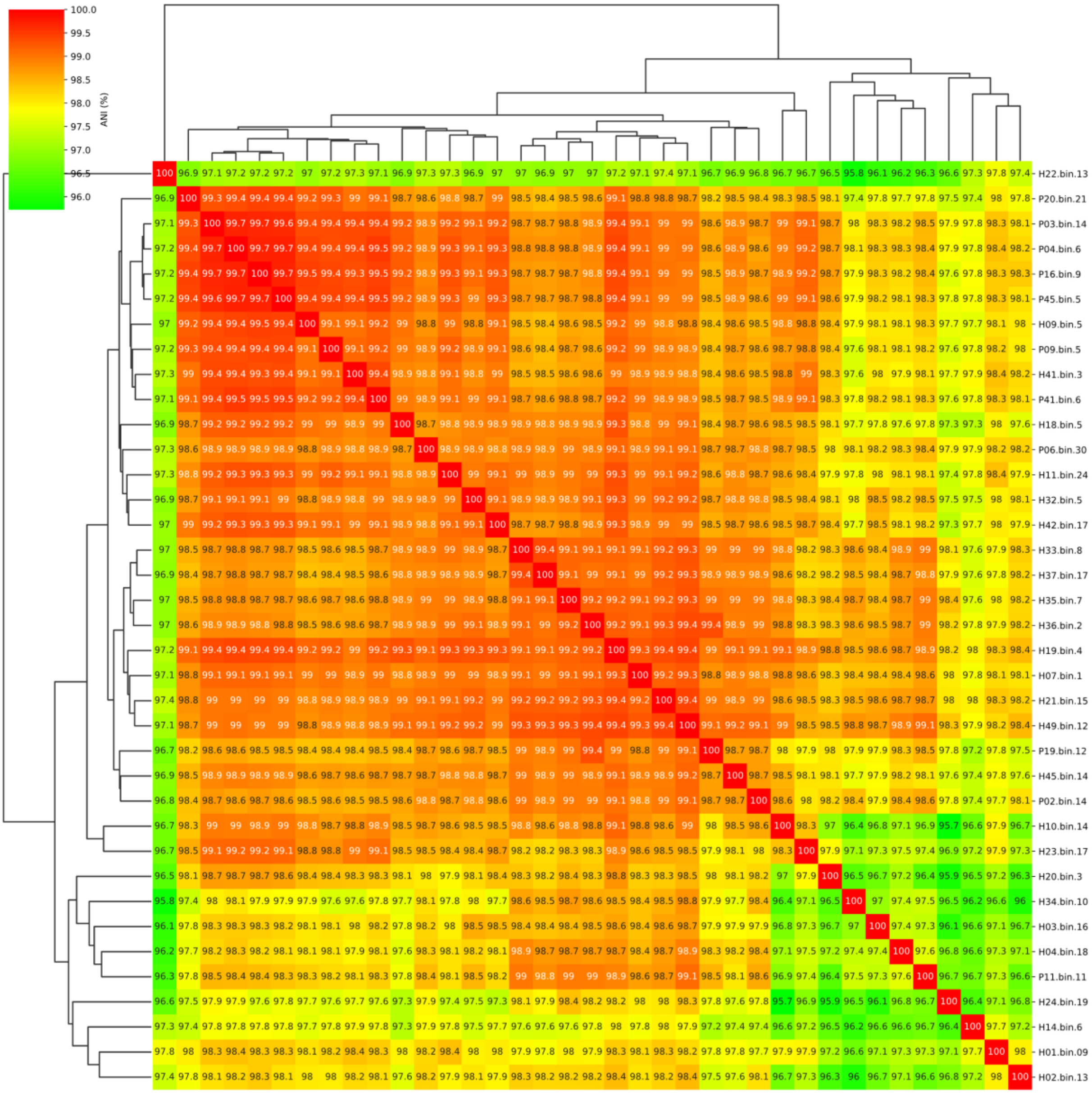

Supplementary Figure 7. ANI cluster heatmap of *E.coli* MAGs recovered

References

1. Nayfach, S. and K.S. Pollard, Average genome size estimation improves comparative metagenomics and sheds light on the functional ecology of the human microbiome. Genome Biology, 2015. 16(1): p. 51.
2. Vincent D Blondel, Jean-Loup Guillaume, Renaud Lambiotte, Etienne Lefebvre, Fast unfolding of communities in large networks, in Journal of Statistical Mechanics: Theory and Experiment 2008 (10), P1000
